# The role and therapeutic potential of itaconate in lung disease

**DOI:** 10.1186/s11658-024-00642-1

**Published:** 2024-10-01

**Authors:** Ruyuan He, Yifan Zuo, Ke Yi, Bohao Liu, Congkuan Song, Ning Li, Qing Geng

**Affiliations:** 1 Department of Thoracic Surgery, Renmin Hospital of Wuhan University, Hubei Province, 99 Zhangzhidong Road, Wuhan, 430060 China; 2 Department of Thoracic Surgery, Jilin University, Changchun, China

**Keywords:** Itaconate, Metabolite, Inflammation, Immunometabolism, Macrophage, Lung diseases

## Abstract

Lung diseases triggered by endogenous or exogenous factors have become a major concern, with high morbidity and mortality rates, especially after the coronavirus disease 2019 (COVID-19) pandemic. Inflammation and an over-activated immune system can lead to a cytokine cascade, resulting in lung dysfunction and injury. Itaconate, a metabolite produced by macrophages, has been reported as an effective anti-inflammatory and anti-oxidative stress agent with significant potential in regulating immunometabolism. As a naturally occurring metabolite in immune cells, itaconate has been identified as a potential therapeutic target in lung diseases through its role in regulating inflammation and immunometabolism. This review focuses on the origin, regulation, and function of itaconate in lung diseases, and briefly discusses its therapeutic potential.

## Introduction

Lung diseases remain prevalent clinical conditions with high morbidity and mortality rates. However, targeted drugs are limited to a few diseases, and for most lung conditions, medical management primarily aims at symptom relief and control of disease progression [1–3]. The development of many lung diseases can be attributed to tissue inflammation and oxidative stress injury, whether due to infections or malignancies [2, 4]. An impaired balance between inflammation and anti-inflammation, along with oxidative stress, has been identified as a hallmark in most pulmonary diseases, including lung cancer, acute and chronic lung injury induced by infection or physical/chemical exposure, and pulmonary fibrosis. Despite the varying pathologies, the immune system plays a significant role in these lung diseases. The immune response in lung disease is characterized by altered metabolism in different cellular components. For example, glycolysis in typical M1 macrophages has been demonstrated to be an important initiator and promoter of inflammation.

Given the recognition of the immune system as a key regulator in lung disease, immune metabolism reprogramming has emerged as a potential therapeutic target. The altered metabolism in immune cells, as a reaction and adaptation to both external and internal stimuli, characterizes their functional phenotype. For example, the Warburg effect, which involves increased glucose consumption for the production of biomacromolecule precursors and nicotinamide adenine dinucleotide phosphate (NADPH), occurs during acute stimulation, ensuring cellular survival and proliferation under external stress exposure. In contrast, fatty acid oxidation and the classical tricarboxylic acid (TCA) cycle maintain metabolism in quiescent cells. Interestingly, both metabolites and enzymes involved in these processes can regulate immunometabolism and immune function in return, such as nitric oxide (NO), arginine, and glutamine, revealing a complex interaction. Consequently, efforts have been made to identify the therapeutic potential of immunometabolism regulation in lung disease. For instance, the protective effect of dexamethasone in asthma is associated with downregulation of pyruvate dehydrogenase kinase, glutaminase, and fatty acid synthase [5]. Potential immunometabolic targets in some lung diseases have been previously reported, including the NOD-like receptor protein 3 (NLRP3) inflammasome, the cGAS-STING axis, and beta-hydroxybutyrate [6]. As a metabolite from TCA cycle, itaconate has emerged as a novel and potential metabolic target.

With the onset of the coronavirus disease 2019 (COVID-19) pandemic, lung diseases such as acute lung injury and pulmonary fibrosis have garnered increased attention. Lung diseases are among the leading causes of death globally and significantly impact the quality of life. Itaconate, a macrophage metabolite that has emerged in recent years, has been shown to possess anti-inflammatory and anti-oxidative stress effects, as well as effective immunometabolism regulating functions. This review focuses on the precise anti-inflammatory and anti-oxidative stress functions of itaconate and its exogenous derivatives in various lung diseases, along with their potential mechanisms. By summarizing recent studies primarily on pulmonary inflammatory diseases, it is demonstrated that both itaconate and its exogenous derivatives have anti-inflammatory and antioxidant effects, offering significant pharmaceutical potential.

## The origin and exploration of itaconate

Itaconate was first synthesized by Jean Louis Lassaigne in 1836 during his study of the thermal decomposition of citric acid [7]. Initially, it was used for industrial polymer synthesis until its role in the TCA cycle was investigated in the twentieth century [8]. While itaconate is a potential intermediate in central metabolism, it cannot support respiration in the same way as succinate or malate [8]. In 2004, Sakai and colleagues reported that itaconate induced suppressed glycolysis levels in liver cells, supporting its function as a metabolism regulator [9]. The presence of itaconate in mammalian cells was first observed in 2011 [10], and metabolite profiling revealed increased levels of itaconate in macrophages during lipopolysaccharide (LPS) stimulation [11]. In 2013, Michelucci and colleagues demonstrated that itaconate is endogenously produced in macrophages by the mammalian *cis*-aconitate decarboxylase (CAD), also known as immune response gene 1 protein (IRG1), which encodes aconitate decarboxylase 1 (ACOD1) [12]. Previous studies of IRG1 had identified it as one of the most highly upregulated genes under pro-inflammatory conditions, highlighting its crucial role in the immune response [13, 14]. Thus, IRG1 links cellular metabolism with immune defense by catalyzing the production of itaconate [12]. Increasing evidence from subsequent studies suggests that itaconate has important anti-inflammatory and antioxidant effects in mammals [15, 16]. Many efforts have been made to explore the mechanisms of itaconate function, and the main targets of itaconate that have been reported are summarized in Table 1. Itaconate has been reported as a succinate dehydrogenase (SDH) inhibitor [17, 18] and can inhibit mitochondrial substrate-level phosphorylation [19]. Further studies have shown that itaconate can regulate nuclear factor erythroid 2-related factor 2 (Nrf2) signaling, the IκBζ-ATF3 axis, and NLRP3 inflammasome activation [20–22]. Fructose-bisphosphate aldolase A, Janus kinase 1 (JAK1), TET DNA dioxygenases, and transcription factor EB (TFEB) have also been identified as itaconate targets [23–26].

**Table 1:** Currently reported targets and effects of itaconate

Itaconate target and effect	Study
Itaconate inhibits fructose 2,6-bisphosphate synthesis	2004, Sakai et al.
Itaconate abolishes mitochondrial substrate-level phosphorylation	2016, Németh et al.
Itaconate inhibits succinate dehydrogenase	2016, Cordes et al.; 2016, Lampropoulou et al.
Itaconate activates Nrf2 Signaling	2018, Tang et al.
Itaconate inhibits IκBζ–ATF3 axis	2018, Bambouskova et al.
Itaconate inhibits fructose-bisphosphate aldolase A	2019, Qin et al.
Itaconate inhibits NLRP3 inflammasome activation	2020, Hooftman et al.
Itaconate inhibits TET DNA dioxygenases	2022, Chen et al.
Itaconate inhibits Janus kinase 1	2022, Runtsch et al.
Itaconate alkylates TFEB	2022, Zhang et al.

## The regulation of itaconate and ACOD1

Itaconate, derived from citrate, is primarily produced by macrophages and myeloid cells under M1 polarization conditions [27]. Immune organs such as lymph nodes and spleen have the highest concentrations of itaconate [28]. The synthesis of this immune metabolite is induced by the enzyme CAD, encoded by ACOD1/IRG1, which catalyzes the decarboxylation of *cis*-aconitate to itaconate. This enzyme has low basal expression under normal conditions, but its expression increases upon exposure to environmental contaminants or infections [12, 29–31]. The anaplerotic TCA cycle in active macrophages produces high levels of itaconate [32]. As the only enzyme capable of catalyzing itaconate synthesis, IRG1’s expression is tightly regulated, though its regulatory mechanisms are still not fully understood. Interferon regulatory factor 1 is the most important transcriptional regulator of IRG1 [33]. TFEB, a lysosomal biogenesis factor activated by bacterial stimuli, drives IRG1 expression and itaconate synthesis in macrophages [34]. Itaconate in macrophages can induce TFEB nuclear translocation and activation by inducing TFEB alkylation [26]. The stimulator of interferon response cGAMP interactor 1 is also essential for LPS-induced IRG1 expression [35]. Additionally, NO and isocitrate dehydrogenase activity regulate the TCA cycle and itaconate production, thereby modulating respiratory function. A deficiency in NO in macrophages leads to increased itaconate accumulation and elevated interleukin-1β (IL-1β) level [36].

The expression of IRG1 can be regulated by several signaling pathways. Activated nuclear factor kappa-B (NF-κB) signaling can induce IRG1 expression, leading to the accumulation of itaconate in macrophages [37]. Increased IRG1 expression was also observed in A20-deficient macrophages [38]. Since the ubiquitin enzyme A20 can inhibit NF-κB, IRG1 might be upregulated by A20 through its inhibition of NF-κB signaling. The IRF9–IRG1 pathway was also previously reported, showing that decreased IRF9 levels, due to miR93, lead to the inhibition of IRG1 [39]. Other novel signaling pathways, such as the NOTCH4/GATA4/IRG1 axis, the SR-A1/STAT3/IRG1 axis, and TFEB/IRG1, have also been demonstrated as potential regulators of itaconate [34, 40, 41]. Additionally, specific knockout of peroxisome proliferator-activated receptor gamma (PPARγ) in macrophages leads to increased IRG1 expression, suggesting a potential regulatory effect of PPARγ on itaconate production [42]. IFN-I decreases itaconate production through IRG1 inhibition induced by IL-10 [43].

**Fig. 1:**
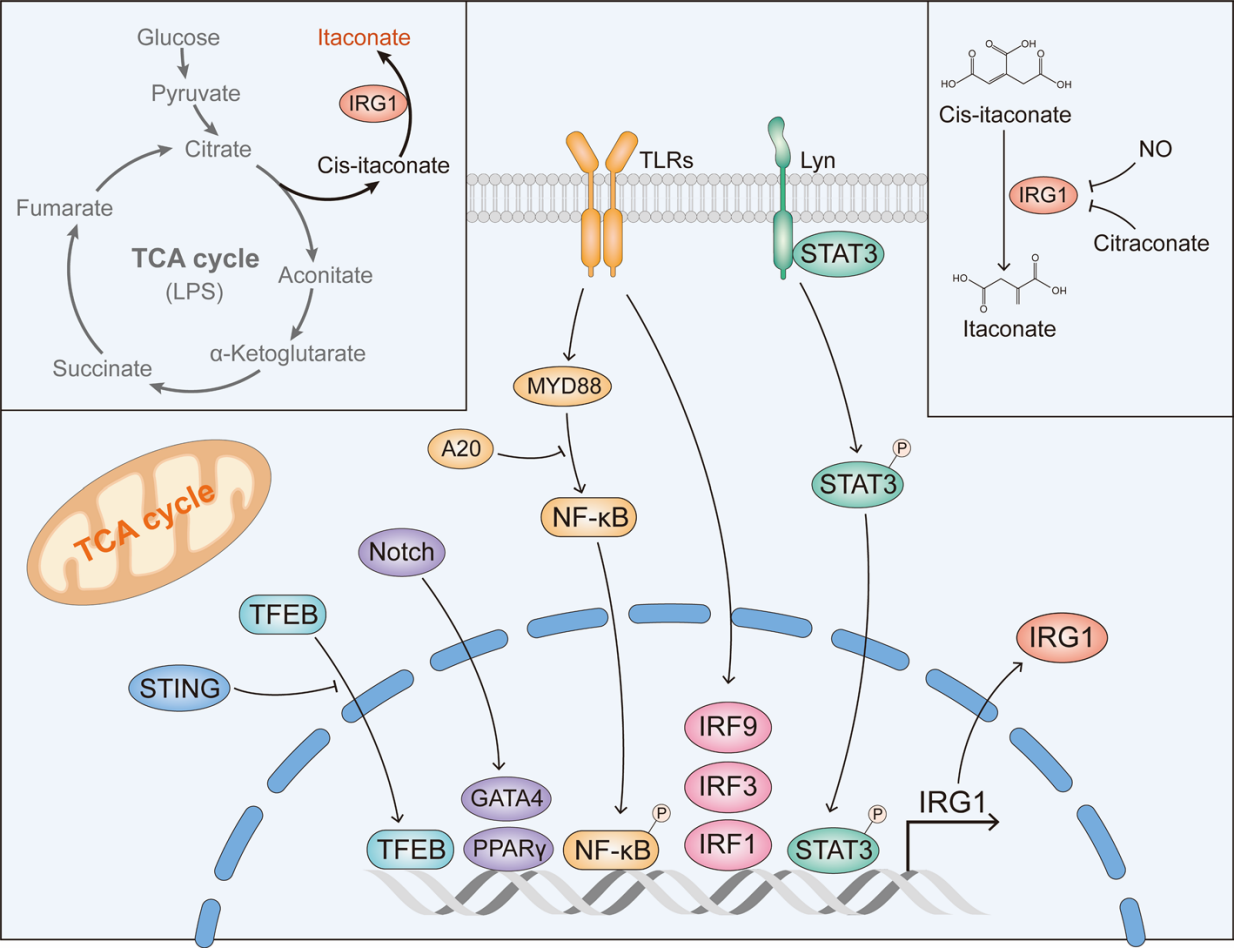
The regulation of itaconate and IRG1. A typical upregulation of IRG1 occurs in macrophages after the LPS stimuli. The *cis*-aconitate are transferred and readjusted from TCA-cycle, leading to the accumulation of itaconate. The IRG1 expression can be regulated by many pathways, such as Norch/GATA4, NF-κB, STING, or phospho-STAT3; meanwhile, itaconate production can be suppressed by NO and citraconate. Pointed arrows and blunt head arrows indicate promotion and inhibition, respectively

An interesting fact is that, although IRG1 can be upregulated by activated NF-κB signaling, the consequent accumulation of itaconate promotes Nrf2/HO-1 signaling and inhibits signal transducer and activator of transcription 3 expression, thereby suppressing NF-κB signaling [44, 45]. Additionally, reactive oxygen species (ROS) production in a murine colitis model is decreased by 4-octyl itaconate (4-OI), which inhibits the activation of MAPK/NF-κB signaling [46]. The regulation of itaconate and IRG1/ACOD1 is presented in Fig. 1.

## The function of itaconate

### Itaconate regulates immunometabolism and inflammation

IRG1 encodes a mitochondrial metabolic enzyme that catalyzes itaconate production. Under pathological conditions, such as LPS stimulation, the upregulation of the IRG1 enzyme stimulates the conversion of *cis*-aconitate to itaconate in the TCA cycle [26]. Previous studies have highlighted that itaconate functions as an important anti-inflammatory metabolite, acting as an SDH inhibitor, Nrf2 regulator, and electrophilic stress regulator [32]. Itaconate is partially upregulated by interferon-β (IFN-β) and promotes an anti-inflammatory response through Nrf2 activation and NLRP3 inhibition [16, 47]. Unmodified natural itaconate inhibits inflammasome activation and increases LPS-induced production of IFN-β in macrophages [48]. The anti-inflammatory effect of itaconate is related to SDH inhibition and changes in mitochondrial respiration in active inflammatory macrophages. The endogenous accumulation of itaconate in macrophages leads to the accumulation of succinate, a metabolite associated with the metabolic reprogramming of immune cells [17, 18]. Itaconate is metabolized into itaconyl-CoA, which modulates methylmalonyl-CoA-dependent branched-chain amino acid metabolism, altering the balance of CoAs and fatty acid diversity [49]. Additionally, itaconate stabilizes the expression of carnitine palmitoyltransferase 1A (CPT1A) by interfering with CPT1A ubiquitination, thereby enhancing lipid clearance and modulating lipid metabolism [50]. These results demonstrate that itaconate is a key regulator in TCA cycle remodeling.

4-Octyl itaconate (4-OI), a cell-permeable derivative of itaconate, blocks the activation of the NLRP3 inflammasome and IL-1β release as well as inhibits STING expression and PI3K/Akt signaling [22, 51, 52]. Additionally, itaconate derivatives can directly modify and inhibit JAK1 [25]. Another membrane-permeable itaconate derivative, dimethyl itaconate (DI), can suppress the secretion of cytokines IL-1β and C–C motif chemokine ligand 2 in epithelial cells and reduce macrophage recruitment. This leads to alleviation in ulcerative colitis in a mouse model and promotes T cell differentiation [53]. Exposure to LPS treatment upregulates IRG1 expression in macrophages, resulting in increased itaconate levels. This increase in itaconate levels may cause mitochondrial substrate-level phosphorylation loss due to potential CoA trapping [19]. The fluctuations in IRG1 and itaconate levels in macrophages might reduce the survival of infectious microbes or impair macrophage function under hypoxic stress [19]. The absence of IRG1 expression in myeloid cells contributes to the activation of NF-κB signaling, leading to excessive neutrophil recruitment and increased mortality in mice during infection. This excessive inflammation can be mitigated by exogenous itaconate supplementation [54]. Itaconate and DI can interfere with IκBζ signaling in inflammatory responses by increasing ATF3 expression [21]. Additionally, itaconate can target specific pathogens to inhibit bacterial growth [53]. By disrupting the Fe–S cluster structure within aconitase, itaconate inhibits its activity and regulates iron metabolism in macrophages [55]. Itaconate directly binds to teneleventranslocation-2, inhibiting its activity, which results in decreased 5hmC levels and protects mice from LPS-induced injury [24]. Increased itaconate levels have also been reported to inhibit TET DNA dioxygenases, thereby suppressing inflammation and reducing immune cell infiltration into tumors [37]. Structurally similar to α-ketoglutaric acid (α-KG), a dicarboxylic acid with 4- or 5-carboxylate groups, itaconate can act as an α-KG antagonist. This similarity allows ITA to selectively inhibit TET enzymes and suppress inflammatory responses. To further explore downstream pathways, Chen et al. treated TET2/TET2-mutant BMDM cells with or without 4-OI under LPS stimuli. Gene expression profiling revealed that 607 genes were downregulated in TET2-mutant cells, with these genes being enriched in those involved in innate immunity and inflammatory response. Additionally, the results suggested that IκBζ, a transcriptional regulator of selective NF-κB target genes, is encoded by Nfkbiz [24]. Despite its significant anti-inflammatory effects, high doses of itaconate can induce apoptosis and IL-1β release [56].

Itaconate also inhibits virus replication by suppressing SDH activity, which is associated with changes in cellular metabolism [57]. Moreover, itaconate can inhibit fumarate hydratase activity, leading to the accumulation of fumarate and succinate, which are crucial for intracellular energy metabolism [58]. In LPS-activated RAW264.7 cells, itaconate reduces the levels of glycolytic intermediates that are elevated in metabolically reprogrammed macrophages [59]. Itaconate inhibits fructose-bisphosphate aldolase A and glyceraldehyde 3-phosphate dehydrogenase (GAPDH), impairing glycolysis and consequently attenuating the inflammatory response in macrophages [23, 60].

### Itaconate regulates oxidative stress

Itaconate and its analogs have been shown to influence the oxidation–reduction process enhance the expression of antioxidant genes, indicating its potential role in regulating oxidative stress [61, 62]. Itaconate has been reported to modulate mitochondrial ROS production in macrophages [63], and supplementation with the itaconate analog 4-OI can restore mitochondrial redox balance [64]. Upon exposure to endotoxin, IRG1 expression is markedly increased, leading to elevated A20 expression through ROS production in macrophages, which enhances endotoxin tolerance [65]. ROS production by IRG1 is regulated by the pentose phosphate pathway [66]. Itaconate can activate Nrf2 signaling [67, 68]. Itaconate alleviates hepatic ischemia–reperfusion injury by activating the Nrf2-antioxidant pathway, thereby protecting hepatocytes from oxidative stress damage [69]. Additionally, itaconate can regulate the Nrf2-mediated antioxidant response by suppressing fumarate hydratase activity [58]. In the cerebral ischemia–reperfusion model, itaconate induces a cellular antioxidant response and modulates brain redox metabolism, which protects mitochondrial function, improves brain function, and reduces mortality following reperfusion injury [70].

### Itaconate regulates cell death

**Fig. 2:**
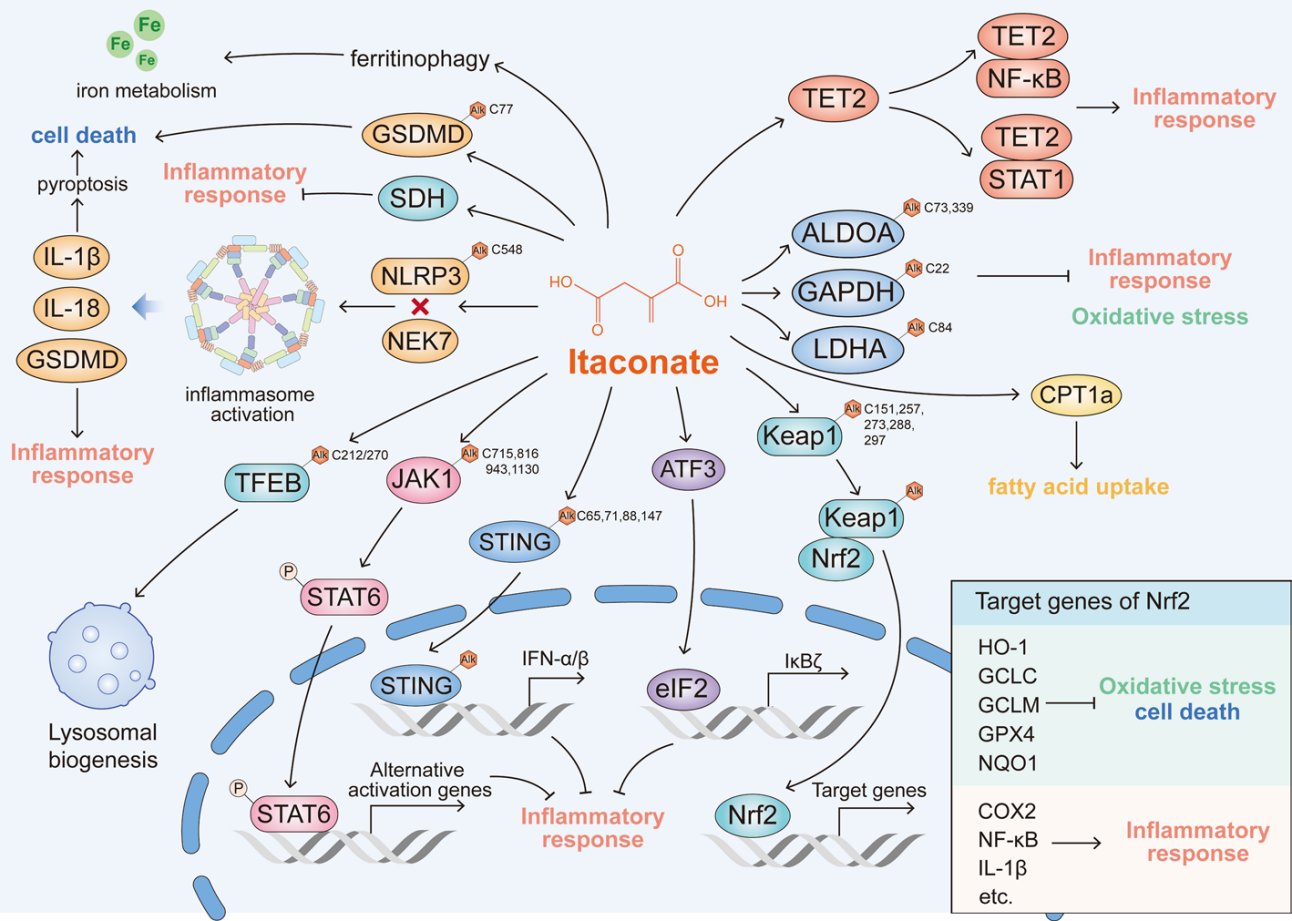
How itaconate regulates immunometabolism, inflammatory response, oxidative stress, and cell death. As a typical immune metabolite that accumulated in LPS-activated macrophages, itaconate is a crucial anti-inflammatory metabolite that acts via different manners. Nrf2-dependent anti-inflammatory function is one main target of itaconate. Itaconate can also inhibit NLRP3 activation, SDH activity, and cGAS-STING-IRF3 pathway. Itaconate interferes with CPT1A ubiquitination to stabilize CPT1A expression. Through this complex regulatory network, itaconate exquisitely models the metabolic alteration and inflammatory response to limit inflammation. Pointed arrows and blunt head arrows indicate promotion and inhibition, respectively

IRG1 is known to be associated with tumor cell proliferation, migration, and invasion, and is highly expressed in tumor cells [45]. Itaconate also increases the expression of hypoxia-inducible factor 1 targets, such as Hk2 and Vegfa [71]. In sepsis-induced mouse models, itaconate elevates Nrf2 levels and protects THP-1 cells from ferroptosis [72], while preserving mitochondrial function and inhibiting the cGAS-STING-IRF3 pathway [73]. Additionally, itaconate modulates LPS-induced pyroptosis by preventing caspase-1 activation and promoting NLRP3 inflammasome tolerance, thus mitigating tissue damage and preventing pyroptotic cell death [74]. Conversely, 4-OI has been reported to activate ferritinophagy and induce ferroptosis in retinoblastoma cells [75]. Itaconate thus appears to suppress cell death in normal cells while inducing ferroptosis in specific tumor cells through different mechanisms, highlighting its complex role in the cell cycle. Figure 2 summarized the known mechanisms by which itaconate regulates immune function, oxidative stress and cell death.

## The role of itaconate in lung disease

### Acute lung injury

Acute lung injury (ALI) is a critical clinical condition characterized by its rapid onset and high mortality rate, making it one of the leading causes of death in critically ill patients. ALI is characterized by impaired vascular endothelial and alveolar epithelial functions, leading to inflammatory infiltration, pulmonary edema, and arterial hypoxemia [76]. The progression of ALI is primarily driven by inflammation and oxidative stress. Activated platelets contribute to the pathogenesis of ALI by promoting innate immune responses, such as neutrophil recruitment and the production and secretion of proteases and toxic mediators in the lungs [77]. Concurrently, various inflammatory stimuli trigger pulmonary endothelial cells, alveolar cells, airway epithelial cells, and alveolar macrophages to produce ROS and reactive nitrogen species, which further exacerbate lung damage [78]. Currently, managing inflammation is the main therapeutic approach for ALI. Itaconate, with its anti-inflammatory and antioxidant properties, may offer potential medicinal value for treating this condition.

Itaconate exerts anti-inflammatory effects through SDH inhibition, Nrf2 activation, and reduction of pro-inflammatory cytokine production [79]. In an LPS-induced ALI model, the administration of 4-OI significantly reduces the production of pro-inflammatory mediators and ROS [72]. The protective effects of 4-OI have also been observed in two additional models: the LPS-induced murine acute kidney injury model and the microglia inflammation model [80, 81]. Similarly, in a methicillin-resistant *Staphylococcus aureus*-induced ALI model, 4-OI administration improved survival, mitigated pathological damage, and inhibited neutrophil infiltration [82]. Furthermore, 4-OI reduces LPS-induced NLRP3 inflammasome activation and interleukin-1β (IL-1β) secretion [83]. This effect is attributed to the alkylation-mediated dissociation of NLRP3 from NEK7 [22]. Additionally, itaconate reconstitution restores NLRP3 inflammasome tolerance in IRG1-deficient macrophages [74]. DI has been shown to inhibit LPS-induced microglia activation via the Nrf2/HO-1 signaling pathway [84]. Collectively, these studies suggest that itaconate and its analogs offer significant protective effects and have considerable clinical potential in managing inflammatory responses.

In addition to inflammatory infiltration, ALI causes redox imbalance, oxidative damage, and DNA damage, which collectively lead to lung cell death and dysfunction. Oxidative and antioxidant pathways are regulated by various external stimuli. 4-OI promotes the nuclear transport of Nrf2, a crucial endogenous antioxidant transcription factor, thereby enhancing the antioxidant response downstream of the Nrf2 signaling pathway [44]. In the context of oxidative stress in vivo, lipids are also subject to peroxidation. As previously reported, 4-OI alleviates ALI by inhibiting glutathione peroxidase 4-dependent lipid peroxidation, which is achieved through increased Nrf2 accumulation and activation [72]. Similarly, when the Nrf2 signaling pathway is blocked, the protective effects of 4-OI are significantly diminished in chondrocytes and mouse arthritis models. The overaccumulation of neutrophil extracellular traps (NETs)—which form specialized bactericidal structures following necrosis or apoptosis—can cause tissue damage. 4-OI inhibits NET formation in mouse neutrophils via the Nrf2/HO-1 pathway, thereby contributing to a favorable long-term prognosis [85]. Additionally, itaconate enhances the resistance of pulmonary microvascular endothelial cells to inflammation and mitochondrial oxidative stress by activating the Nrf2 pathway [86]. Itaconate also promotes the nuclear translocation of TFEB to regulate autophagic flux, thus reducing apoptosis [87].

The results of these studies suggest that itaconate has the potential to reduce inflammation and restore redox balance during tissue injury. Specifically, itaconate may exert its therapeutic effects through multiple pathways. For instance, reduced ROS production may influence ROS-mediated PI3K/Akt/NF-κB signaling, potentially contributing to the alleviation of LPS-induced lung inflammation by itaconate. Additionally, itaconate is known to inhibit TET enzymes, which may also partially explain its benefits in managing inflammation. Another challenge in using itaconate as a therapeutic agent for lung injury is coordinating its effects with the response to infecting pathogens. Since pathogens may also respond to changes in environmental itaconate levels, further studies are needed to determine whether pathogens adapt to exogenous itaconate supplementation and how this adaptation might affect disease progression. The antibacterial, anti-inflammatory, and antioxidant properties of itaconate result from the interaction and coordination of multiple mechanisms, reflecting its complexity and versatility as a regulator of immune metabolism rather than a mere metabolite.

### Respiratory infection

As the organ responsible for gas exchange with the external environment, the lungs are naturally exposed to a wide range of potential pathogens, including bacteria, viruses, and fungi. Itaconate, as a potential immunomodulator, has been explored for its therapeutic potential in various pathogen infections. Gu et al. reported that dimethyl itaconate (DI) protects against fungal keratitis by activating the Nrf2/HO-1 signaling pathway and inhibiting the growth of *Aspergillus fumigatus* [88]. However, there is limited evidence that itaconate protects hosts from fungal infections. Given that fungi are a significant source of industrial itaconate [89], understanding itaconate metabolism in fungal infections is essential. In influenza A virus infection, itaconate and its analogs reduce inflammation and inhibit viral replication [90–92]. Notably, itaconate inhibits virus replication not through Nrf2-independent mechanisms but by directly interfering with the nuclear export of viral ribonucleoprotein complexes [91, 92]. Another area of interest is the interaction between bacteria and itaconate, as itaconate can directly impact bacterial cells. itaconate enhances NADPH oxidase activity, leading to increased ROS production and inhibition of bacterial growth [93]. The antimicrobial capacity of itaconate is also associated with its disruption of central carbon metabolism [94, 95]. Itaconate can covalently modify the active-site cysteine of isocitrate lyase, thereby inhibiting the enzyme and suppressing bacterial growth [96, 97]. However, bacteria can adapt to changes in environmental itaconate levels in several ways. For example, *Staphylococcus aureus* experiences ammonium starvation under itaconate stress [98], and the LysR-type transcriptional regulator RipR protects Gram-negative bacteria from itaconate challenges [99].

The novel coronavirus epidemic has resulted in over 50 million deaths and countless infections worldwide. As a disease that has garnered significant attention in recent years, numerous studies have explored the relationship between itaconate and novel coronaviruses. Severe acute respiratory syndrome coronavirus 2 (SARS-CoV-2) is a positive-stranded RNA beta-coronavirus that facilitates receptor recognition and membrane fusion through the spike glycoprotein (S protein) on the surface of the virion. This interaction with the host receptor, angiotensin-converting enzyme 2 (ACE2), mediates viral entry and can lead to severe respiratory syndrome [100]. SARS-CoV-2 drives a cytokine storm and causes immunosuppression, resulting in a hyperinflammatory state that contributes to multi-organ failure, particularly respiratory failure due to acute respiratory distress syndrome (ARDS) [101, 102]. Given that cytokine storms and oxidative stress are major contributors to ARDS during respiratory viral infections, anti-inflammatory and antioxidant therapies have emerged as effective therapeutic strategies.

Itaconate is a potential agent in the treatment of COVID-19 due to its antioxidant and immune metabolism-regulating effects. Metabolic remodeling plays a crucial role in viral infections, as host metabolism is integral to both the host immune response and viral propagation [103]. Itaconate regulates metabolic remodeling by modulating electron transport chain flux and lipid metabolism, which in turn affects macrophage activation and inflammatory responses [18, 103]. Analysis of a publicly available transcriptomic dataset from SARS-CoV-2 patient lung biopsies revealed that the Nrf2 antioxidant gene expression pathway is inhibited. David et al. found that in vitro administration of itaconate can induce cellular antiviral programs via a type I interferon-independent pathway [104]. Additionally, Nrf2 significantly inhibits SARS-CoV-2 replication by activating downstream HMOX1 to produce biliverdin, which scavenges ROS [104]. However, SARS-CoV-2 interacts with the catalytic domain of the NAD-dependent deacetylase sirtuin 1 (SIRT1) via the nonstructural viral protein NSP14, thereby inhibiting the activation of the Nrf2/HMOX1 pathway [105]. Similarly, decreased levels of Nrf2 have been observed in pediatric patients infected with SARS-CoV-2, resulting in reduced total antioxidant status and increased oxidative stress, leading to tissue damage.

Physiologically, the presence of ROS leads to the dissociation of the Kelch-like ECH-associated protein 1 (Keap1)-Nrf2 complex, allowing Nrf2 to migrate to the nucleus. This migration triggers antioxidant responses, enhancing protection against inflammation. During viral infections, such as SARS-CoV-2, inflammatory processes and oxidative stress in epithelial and endothelial cells activate the transcription factor Nrf2, which similarly protects cells from oxidative stress and inflammation. Concurrently, SARS-CoV-2 infection is associated with alterations in lipid metabolism. The Spike protein on the surface of SARS-CoV-2 impairs lipid metabolism and autophagy pathways in host cells by upregulating Nrf2, leading to increased siderosis and heightened susceptibility to lipid toxicity [106]. This spike-induced impairment in lipid metabolism can be inhibited by Nrf2 inhibitors [106]. However, Nrf2 deficiency can upregulate angiotensin-converting enzyme 2 (ACE2), facilitating the entry of severe acute respiratory syndrome coronavirus 2 (SARS-CoV-2) into respiratory cells [107], indicating the complex role of Nrf2 in SARS-CoV-2 infection.

The evidence highlights the complexity of using itaconate in the treatment of SARS-CoV-2. As an Nrf2 agonist, itaconate may increase cellular susceptibility to lipid toxicity induced by the spike protein. However, itaconate also inhibits SARS-CoV-2 replication through the Nrf2/HMOX1 pathway. Recently, it has been reported that 4-OI can block inflammation-associated coagulation by inhibiting type I interferon signaling and the release of tissue factor [108]. This finding offers a novel perspective on the therapeutic role of itaconate in managing inflammation.

### Pulmonary fibrosis

Pulmonary fibrosis is a severe disease of unknown etiology characterized by excessive deposition of extracellular matrix and destruction of lung structure, leading to impaired gas exchange [109]. Studies have highlighted the role of alveolar macrophages (AMs) in regulating the pathogenic mechanisms underlying idiopathic pulmonary fibrosis (IPF), including lung defense, repair, surfactant handling, and inflammatory responses [110]. Given that itaconate is a major physiological regulator of global metabolic rewiring and the effector function of inflammatory macrophages, it holds potential therapeutic promise for influencing the development and progression of pulmonary fibrosis.

The expression of ACOD1 in alveolar macrophages (AMs) and itaconate levels in bronchoalveolar lavage fluid were both reduced in patients with idiopathic pulmonary fibrosis (IPF), indicating a significant alteration in the ACOD1/itaconate axis in fibrotic lung tissue [111]. In a bleomycin-induced mouse model of pulmonary fibrosis, specific knockout of ACOD1 resulted in exacerbation of the disease. Notably, adoptive transfer of wild-type monocyte-derived AMs into ACOD1-deficient mice restored the disease phenotype induced by bleomycin exposure. Additionally, the antifibrotic effects of inhaled itaconate were demonstrated in mice, confirming that itaconate is crucial for controlling the severity of pulmonary fibrosis [111]. Itaconate has also been shown to activate the Nrf2 pathway, which reduces the expression of thioredoxin interacting protein in the lung interstitium and mitigates fibroblast–myofibroblast differentiation (FMD)—a key cell phenotype in the development and progression of pulmonary fibrosis. Nrf2 inhibits transforming growth factor-β1-induced increases in FMD and ROS, thereby alleviating fibrosis [112]. It can be reasonably inferred that itaconate exerts beneficial effects on pulmonary fibrosis by activating the Nrf2 pathway. The potential mechanisms through which the Nrf2 pathway affects fibrotic disease have been well summarized [113]. As a natural metabolite and Nrf2 agonist, itaconate inhibits the initiation and progression of fibrosis by activating multiple downstream protective proteins via Nrf2, suggesting that itaconate could serve as a novel endogenous antifibrotic agent in the treatment of IPF.

### Chronic obstructive pulmonary disease and asthma

Chronic obstructive pulmonary disease (COPD) is a condition characterized by persistent airflow limitation and is associated with a heightened chronic inflammatory response to irritants, while asthma is primarily associated with lower airway inflammation [1, 114]. Effective management of airway inflammation has been shown to alleviate symptoms in both COPD and asthma. The Nrf2 activator CPUY192018 can inhibit glycolysis and enhance antioxidant responses, thereby ameliorating inflammatory responses. CPUY192018 activates the Nrf2 pathway by disrupting the interaction between Keap1 and Nrf2, thereby reprogramming macrophage metabolism in an Nrf2-dependent manner [115]. Given that immune cells, such as macrophages, play a significant role in COPD pathogenesis [116], itaconate and its derivatives may also offer protective effects as effective Nrf2 activators. Further research is needed to determine whether itaconate administration can alleviate COPD. In asthma, loss of IRG1/itaconate was found to impair mitochondrial function in airway dendritic cells (DCs), leading to enhanced antigen priming. Administration of 4-OI [10 mg/kg intaperitoneally (i.p.)] restored mitochondrial redox balance in DCs and reduced the inflammatory response. Exogenous itaconate administration has been shown to decrease airway inflammation [64]. The inflammatory response is crucial in the pathogenesis of both COPD and asthma, and the anti-inflammatory and antioxidant effects of itaconate may offer novel therapeutic strategies.

### Lung cancer

Lung cancer is a malignant tumor originating from the bronchial mucosa or glands of the lungs, characterized by rapid growth and high morbidity and mortality rates. Over the past 50 years, the incidence and mortality of lung cancer have significantly increased in many countries [117]. The etiology of lung cancer is still not fully understood. Lung cancer induces changes in the tumor microenvironment, including abnormal inflammatory cytokine profiles and oxidative stress. As an effective anti-inflammatory and antioxidant agent, itaconate may offer potential protective effects against the occurrence and progression of lung cancer.

The evidence linking itaconate to cancer is limited, and its role varies across different types of cancer. In some studies, itaconate has demonstrated anticancer effects. For instance, in colorectal cancer, which shares risk factors with lung cancer such as an unhealthy diet, smoking, and chronic inflammation, 4-OI inhibits aerobic glycolysis by targeting GAPDH, thereby promoting cuproptosis [118]. Additionally, DI has been shown to reduce the inflammatory state associated with ulcerative colitis and lower the risk of colitis-associated cancer [53]. In hepatocellular carcinoma, itaconate has exhibited anticancer effects comparable with those of 5-fluorouracil [119]. DI also has demonstrated anti-inflammatory and antitumor effects in human thymic cancer cell lines [120]. However, itaconate may also contribute to cancer progression. In non-small cell lung cancer, Nestin interacts with the Kelch domain of Keap1, leading to the escape of Nrf2 from Keap1-mediated degradation and subsequent promotion of antioxidant enzymes. The modulation of serotonin is emerging as a potential anticancer therapy, given that aberrant cancer antioxidant capacity may drive tumor malignancy. This suggests that the Nestin–Keap1–Nrf2 axis functions as a regulator of cellular redox homeostasis and confers resistance to oxidative stress in non-small cell lung cancer [121]. Itaconate has also been reported to downregulate PPARγ, a tumor suppressor, while upregulating anti-inflammatory cytokines in M2-like macrophages [122]. Furthermore, itaconate may promote tumor growth by suppressing cytotoxic CD8^+^ T cells and enhancing neutrophil resistance to ferroptosis [123, 124]. Itaconate’s role as an immune metabolic component in the crosstalk between tumor-associated macrophages and the tumor microenvironment indicates that dysregulated IRG1 expression may promote tumorigenesis by modulating antitumor immunity [29, 123]. Given the unclear relationship between itaconate and cancer, itaconate may not be a direct therapeutic target for lung cancer. Nonetheless, Wang et al. introduced novel CAR macrophages with enhanced polarization and antitumor functions through ACOD1 depletion [125], suggesting that itaconate might still have potential applications in lung cancer treatment in other contexts.

## The therapeutic potential of itaconate derivatives

Itaconate is a pentacarbon dicarboxylic acid characterized by α,β-unsaturated olefins with mild electrophilic properties [32]. Although itaconate is generally considered a negatively charged polar metabolite with limited cell permeability, it can accumulate intracellularly in macrophages [48]. To overcome the permeability issue, various esterified derivatives of itaconate have been synthesized. Notably, 4-OI and DI are two commonly used derivatives with enhanced membrane permeability. Evidence shows that 4-OI can be converted intracellularly to itaconate by esterase, whereas DI does not methylate to itaconate [60, 126]. Other metabolites, such as fumarate, malonate, succinate, and phosphoenolpyruvate, share structural similarities with itaconate, which contributes to its role as a competitive inhibitor against these similar metabolites. Additionally, isomers of itaconate, including mesaconate and citraconate, exhibit similar immunomodulatory effects [59, 127]. In Table 2, we summarize the main characteristics of the most common itaconate derivatives, including their cell uptake, electrophilicity, and ability to convert to intracellular itaconate [48]. The immunomodulatory functions of itaconate and its derivatives involve several mechanisms, including SDH inhibition, Nrf2 activation, NLRP3 inflammasome inhibition, and glycolysis inhibition [128]. Recently, a novel post-translational modification known as lysine itaconylation has been reported [129], offering new perspectives for further functional studies of itaconate.

**Table 2:** Characteristics of different itaconate derivatives

	Cell uptake ability	Ability to convert to intracellular itaconate	Relative magnitude of electrophilicity
Itaconate	Yes	Yes	Slight
Dimethyl itaconate (DI)	Yes	No	Yes
4-octyl itaconate (4-OI)	Yes	No	Yes
4-monoethyl itaconate (4-EI)	Yes	Few	Slight

**Fig. 3:**
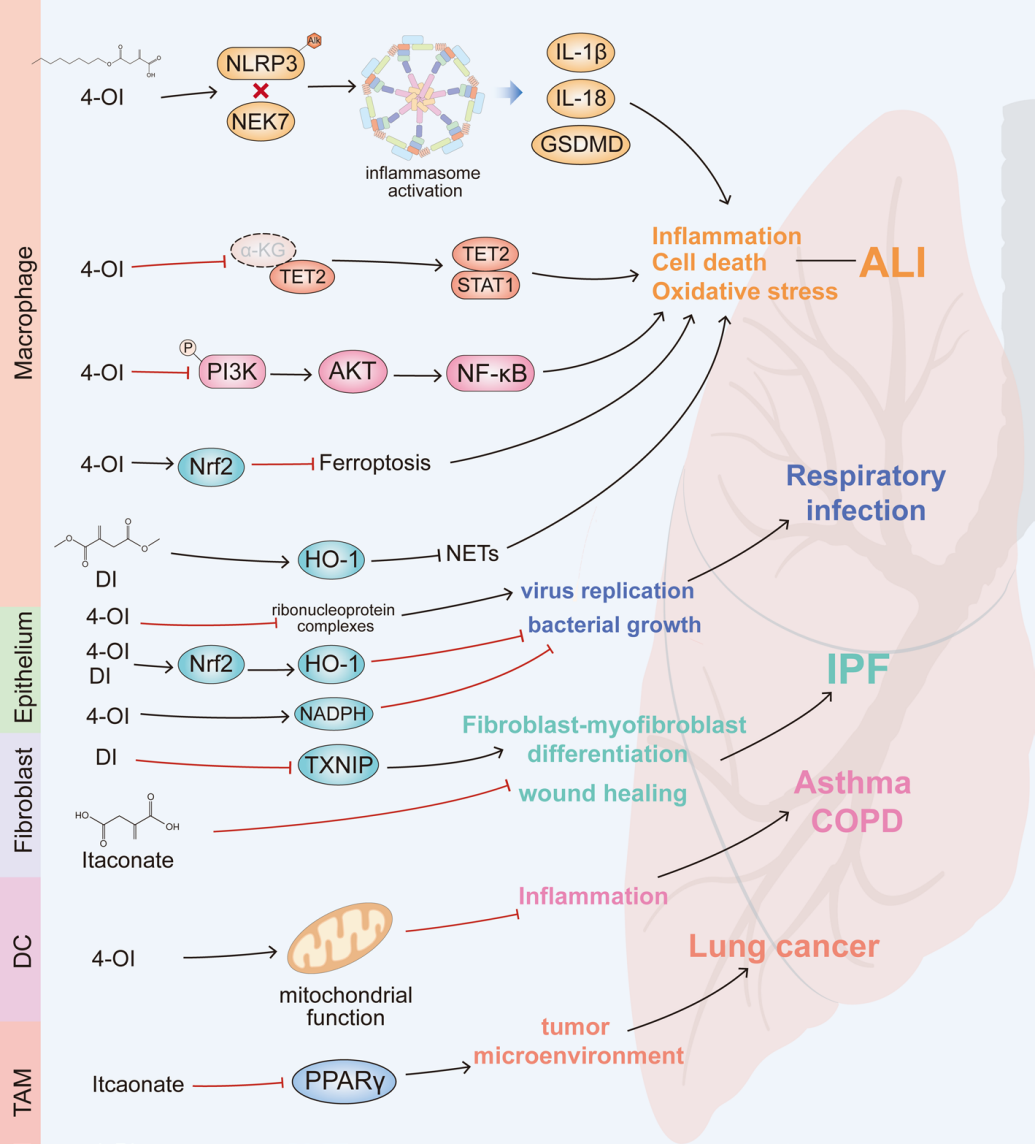
Itaconate and its derivatives can affect different lung diseases via various pathways, such as lung injury, asthma, fibrosis, lung cancer, etc. 4-OI and DI are the most commonly used itaconate derivatives in pulmonary diseases. Most studies focused on macrophages. 4-OI and DI attenuate acute lung injury via several pathways, such as TET2 inhibition, PI3K/AKT/NF-κB signaling inhibition, Nrf2-dependent ferroptosis inhiation or the NLRP3 inflammasome activation. Itaconate may reduce reactive oxygen species production, attenuate inflammatory responses and enhance the antimicrobial innate immunity through alkylation modification and inhibition of succinate dehydrogenase activity. Itaconate can also inhibit viral replication and bacterial growth. The tumor suppressor factor PPARγ can be suppressed by itaconate in tumor-associated macrophages. In addition, itaconate and its derivatives can function in other pulmonary cellular components, including fibroblasts, epithelial cells and dendritic cells by similar effects in macrophages, thus being involved in the pathogenesis of pulmonary fibrosis, lung cancer, and asthma. Black pointed arrows indicate promotion. Red blunt head arrows indicate inhibition

So far, many itaconate derivatives have been tested. 4-OI, a cell-permeable itaconate derivative with an octyl ester tail, has shown potential protective effects in oxidative stress-related diseases [130, 131]. While 4-OI exhibits similar thiol reactivity to itaconate and can potentially be hydrolyzed to itaconate, it does not lead to intracellular accumulation of itaconate in LPS-activated IRG1-deficient macrophages [48]. This suggests that 4-OI may not effectively convert to intracellular itaconate, as itaconate can only arise from the de-esterification of derivatives in these macrophages. Nevertheless, 4-OI remains a functional Nrf2 activator [16]. In contrast, DI enhances LPS-mediated de novo synthesis of itaconate but does not convert to itaconate itself [126]. DI has increased electrophilicity compared with itaconate due to the esterification of the carboxyl group [132], and it is an effective and durable Nrf2 activator. The Nrf2-itaconate axis and antioxidant responses are crucial for pulmonary immune homeostasis. Another therapeutic potential of itaconate is its antiviral property, which can limit viral replication [79]. 4-Ethyl itaconate (4-EI), with a structure similar to DI but lower electrophilicity and higher polarity, cannot inhibit IκBζ. Differences in electrophilicity and other chemical properties among these derivatives can result in variations from the in vivo effects of itaconate. These differences highlight the need for further refinement in pharmaceutical processes to enhance the clinical translation of experimental results. In many lung diseases, itaconate demonstrates anti-inflammatory and anti-oxidative stress functions, which may offer significant implications for the treatment of infectious and inflammatory diseases. In Fig. 3, we summarized the therapeutic potential of itaconate and its derivatives in different lung diseases.

Many efforts have been made to enhance the delivery of itaconate. For instance, itaconate dissolved in saline has been infused before cerebral ischemia reperfusion in mouse models, demonstrating protective effects [70]. Intraperitoneal injection is the most commonly used delivery method in previous studies. Additionally, oropharyngeal administration has emerged as a potentially effective method, particularly for pulmonary diseases. A previous study reported that an inhaled dose of 0.25 mg/kg of itaconate, dissolved in phosphate-buffered saline, can ameliorate bleomycin-induced pulmonary fibrosis in mice [111]. Chen et al. developed a novel mitochondria-targeted supramolecular drug delivery system, 4-OI@Zn-NH-pyr, designed to scavenge both exogenous and endogenous ROS in joint inflammation [133]. Although the efficacy and efficiency of inhalation therapy with itaconate are supported by limited studies and require further validation, oropharyngeal inhalation of itaconate and its derivatives remains a promising and attractive approach. However, several issues need to be addressed before itaconate can be widely applied for lung diseases. While itaconate and its derivatives are functional immunometabolism regulators, they exhibit different modes of action. Both intraperitoneal and oropharyngeal administration methods have shown potential, but further refinement is needed. One concern is that pathogens might exploit the host immune response to sustain infection, which must be considered [134]. For systemic delivery methods, such as intravenous or intraperitoneal injection, itaconate might not accumulate in the lungs as intended, necessitating a targeted delivery vehicle. Inhaled drugs offer lower doses and reduced systemic side effects, but the formulation, dose, and delivery vehicle for itaconate still require further investigation. Despite these challenges, itaconate and its derivatives are promising therapeutic targets for lung diseases. They have been effectively tested in various lung disease models, as summarized in Table 3. Among these studies, LPS was the most common stimulus in animal models [24, 26, 52, 72, 73, 108, 135, 136], while viruses and other pathogens were used in several studies [64, 90, 104, 108, 137–139]. These models primarily assessed therapeutic effects in acute lung infection scenarios, with some research focusing on air pollution [140], chronic lung diseases [112, 141], or ventilator-induced lung injury [131].

**Table 3:** Recent studies involved itaconate and pulmonary disease

Disease	Model	Itaconate formulation		Main mechanisms	References
LPS	Mice	Itaconate	50 mg/kg, i.p.	TET DNA dioxygenases	[24]
LPS	Mice	4-OI	25 mg/kg, i.p.	Nrf2 signaling	[72]
LPS	Mice	4-OI	50/100 mg/kg, i.p.	Nrf2 signaling	[135]
LPS	Mice	4-OI	25 mg/kg, i.p.	PI3K/Akt/NF-κB signaling	[52]
LPS	Mice	4-OI	50 mg/kg, i.p.	cGAS-STING-IRF3 pathway	[73]
LPS	Mice	4-OI	50 mg/kg, i.p.	Gasdermin D-mediated pyroptosis	[136]
LPS, *Staphylococcus aureus*, *E. coli*, SARS-CoV-2	Mice	4-OI, DMF	50 mg/kg, i.p. (4-OI and DMF)	Type I interferon	[108]
LPS	Mice	Depletion of Irg1	Not available	Lysosomal inducer	[26]
*Pseudomonas aeruginosa*	Mice	Depletion of Irg1	Not available	ITA/OXGR1 signaling	[137]
*Staphylococcus aureus*	Mice	Itaconate	Genetic depletion in animal model	Neutrophil glycolysis and NADPH oxidase	[138]
*Brucella*	Mice	Itaconate, 4-OI, DI	Genetic depletion in animal model	Bacterial isocitrate lyase	[139]
SARS-CoV2	Only in vitro	4-OI, DMF	Not available	Nrf2 signaling	[104]
Influenza A virus	Mice	Itaconate, 4-OI, DI	50 mg/kg, i.p.	IFN responses and viral transcription	[90]
Bleomycin	Mice	4-OI	25 mg/kg, i.p.	Nrf2 signaling	[141]
Bleomycin	Mice	DI	50–200 mg/kg, i.p.	Nrf2 signaling	[112]
House dust mite	Mice	Depletion of Irg1	Not available	Antigen presentations by dendritic cells	[64]
Particulate matter	Mice	Itaconate, 4-OI, depletion of Irg1	Genetic depletion in animal model	Nrf2 signaling	[140]
Ventilator-induced lung injury	Mice	4-OI	12.5–50 mg/kg, i.p.	Oxidative stress and NLRP3 activation	[131]

## Future perspective and conclusion

Itaconate and its derivatives have emerged as significant regulators of inflammation and immune metabolism reprogramming. Typically, IRG1, induced by external stimuli like pathogens, leads to itaconate accumulation. This increased itaconate concentration triggers several changes in both immune and nonimmune cells, including SDH inhibition, Nrf2 activation, and electrophilic stress responses.

Given its potential as an anti-inflammatory therapeutic agent, significant efforts have been directed toward exploring itaconate and its derivatives in respiratory diseases, particularly in acute and chronic inflammatory lung conditions such as acute lung injury from sepsis and pulmonary fibrosis. While the efficacy of itaconate and its derivatives has been promising in various animal models, several issues remain unresolved before these compounds can be translated into clinical practice. One critical issue is determining the appropriate doses and concentrations of itaconate and its derivatives for clinical use. Animal studies have employed a wide range of doses, from 12.5 to 200 mg/kg. There is a concern that low doses may offer limited anti-inflammatory effects, while high doses could potentially lead to excessive inhibition of inflammation. Establishing optimal dosing parameters for different lung diseases requires further investigation. Another important consideration is the route of administration. Most studies have used intraperitoneal injection, but intravenous administration might be more suitable for clinical treatment due to its rapid onset and avoidance of first-pass metabolism by the liver. Inhalation is also a viable route, offering the potential for equivalent therapeutic effects at lower doses and bypassing hepatic metabolism. The dosing and formulation of itaconate and its derivatives may vary with different administration routes. Targeted delivery methods, such as modified liposomes, could further enhance the specificity and efficacy of itaconate therapy while minimizing side effects.

In conclusion, itaconate, as a metabolite derived from the TCA cycle, regulates inflammatory responses through various mechanisms and exhibits anti-inflammatory effects with low toxicity. It represents a promising therapeutic target for many lung diseases, particularly those related to inflammation. However, most evidence is derived from animal models, and further research, including clinical studies, is needed to establish the optimal doses, concentrations, and administration routes for clinical application.

## Data Availability

Not applicable.

## Abbreviations

4-OI: 4-Octyl itaconate
ACOD1: Aconitate decarboxylase 1
ARDS: Acute respiratory distress syndrome
α-KG: α-Ketoglutaric acid
CAD: *cis*-aconitate decarboxylase
COPD: Chronic obstructive pulmonary disease
CPT1A: Carnitine palmitoyltransferase 1A
DCs: Dendritic cells
DI: Dimethyl itaconate
FMD: Fibroblast–myofibroblast differentiation
GAPDH: Glyceraldehyde 3 phosphate dehydrogenase
IFN-β: Interferon-β
IRG1: Immune response gene 1
IL-1β: Interleukin-1β
JAK1: Janus kinase 1
Keap1: Kelch-like ECH-associated protein
LPS: Lipopolysaccharide
NADPH: Nicotinamide adenine dinucleotide phosphate
NETs: Neutrophil extracellular traps
NF-κB: Nuclear factor kappa-B
NLRP3: NOD-like receptor protein 3
NO: Nitric oxide
Nrf2: Nuclear factor erythroid 2-related factor 2
IPF: Idiopathic pulmonary fibrosis
PPARγ: Peroxisome proliferator-activated receptor gamma
ROS: Reactive oxygen species
SARS-CoV-2: Severe acute respiratory syndrome coronavirus 2
SDH: Succinate dehydrogenase
TCA: Tricarboxylic acid
TFEB: Transcription factor EB
